# Association of Spermatogenic Failure with the b2/b3 Partial AZFc Deletion

**DOI:** 10.1371/journal.pone.0034902

**Published:** 2012-04-13

**Authors:** Abdelmajid Eloualid, Houria Rhaissi, Ahmed Reguig, Safaa Bounaceur, Brahim El houate, Omar Abidi, Majida Charif, Noureddine Louanjli, Elbakkay Chadli, Abdelhamid Barakat, Anu Bashamboo, Ken McElreavey, Hassan Rouba

**Affiliations:** 1 Human Genetic Unit, Research Department, Pasteur Institute, Casablanca, Morocco; 2 Cytogenetic Unit, Pasteur Institute, Casablanca, Morocco; 3 LABOMAC Laboratory, Casablanca, Morocco; 4 Human Developmental Genetics Unit, Pasteur Institute, Paris, France; 5 Faculty of Sciences Ben M'sik, Casablanca, Morocco; Florida State University, United States of America

## Abstract

Infertility affects around 1 in 10 men and in most cases the cause is unknown. The Y chromosome plays an important role in spermatogenesis and specific deletions of this chromosome, the AZF deletions, are associated with spermatogenic failure. Recently partial AZF deletions have been described but their association with spermatogenic failure is unclear. Here we screened a total of 339 men with idiopathic spermatogenic failure, and 256 normozoospermic ancestry-matched men for chromosome microdeletions including AZFa, AZFb, AZFc, and the AZFc partial deletions (gr/gr, b1/b3 and b2/b3).

AZFa and AZFc deletions were identified in men with severe spermatogenic failure at similar frequencies to those reported elsewhere. Gr/gr deletions were identified in case and control populations at 5.83% and 6.25% respectively suggesting that these deletions are not associated with spermatogenic failure. However, b2/b3 deletions were detected only in men with spermatogenic failure and not in the normospermic individuals. Combined with our previous data this shows an association of the b2/b3 deletion (p = 0.0318) with spermatogenic failure in some populations. We recommend screening for this deletion in men with unexplained spermatogenic failure.

## Introduction

Infertility is defined as the inability to conceive or produce an offspring after one year of unprotected intercourse [1]. It is estimated that infertility affects 10–15% of couples, and roughly half of these cases are due to the male partner [2]. The identification and classification of male infertility requires semen analysis, obtained on at least two separate occasions, and reported according to standard reference values set out by the World Health Organization (1992). Sperm disorders are the most common cause of male factor infertility [3]. Infertile men can present with azoospermia, oligozoospermia, asthenozoospermia, teratozoospermia or by any combination of these.

There are several genes on the Y chromosome necessary for spermatogenesis [4]. Tiepolo and Zuffardi in 1976 identified deletions of the long arm of the Y chromosome associated with spermatogenic failure [5]. Subsequently these deletions were characterised in the euchromatic part of the long Y arm and designated as AZoospermia Factors (AZFa, AZFb and AZFc) [6]. These regions contain several genes or gene families that are expressed in the testis and involved in spermatogenesis [7]. Complete deletions of AZFa, AZFb or AZFc regions are usually accompanied with different histopathological profiles; the AZFa region contains the genes *USP9Y* and *DBY* (also called *DDX3Y*), Protein-encoding gene families on the AZFb region include *RBMY*, *PRY*, and *CDY2* which are expressed only in the testis. The complete AZFc region contains eight gene families (*DAZ, BPY2, CDY1, CSPG4LY, GOLGA2LY, TTTY3, TTTY4,* and *TTTY17*), five of which 5 are expressed only in the testis [4], [5]. Deletions affecting the AZFa region are associated with Sertoli cell only type I syndrome (SCOS type I) that is characterized by the total absence of germ cells in seminiferous tubules and associated with non-obstructive azoospermia. Deletions of the complete AZFb region are usually associated with meiotic arrest, whereas complete deletions of AZFc are associated with variable phenotypes including a significant reduction in sperm count and progressive decline in semen quality [8]. Finally, large deletions that encompass either AZFb+c or AZFa+b+c are associated with a lack of testicular sperm. The most frequently deleted region is AZFc (approximately 60% of cases), followed by deletions of the AZFb and AZFb+c or AZFa+b+c regions (35%), while isolated AZFa deletions are rare (5%) [9].

The precise functional contribution of each of these genes and transcription units to spermatogenesis is unknown [10]. Recently several types of AZFc partial deletions had been identified including the gr/gr, b2/b3 and b1/b3 subdeletions [11]. The relationship between these AZF subdeletions and spermatogenic failure is unclear. The gr/gr deletion is the most commonly detected, indeed, the prevalence of gr/gr deletions varied from 2.1 to 12.5% among all cases, and from 0 to 10.2% among normozoospermic controls [11]. This deletion removes half of the AZFc region including two copies of the *DAZ* gene family (*DAZ1/DAZ2*), one copy of the *CDY1* gene family (*CDY1a*) and one copy of the *BPY2* gene [12]. The deletion has been proposed as a risk factor for reduced sperm counts although not all studies have identified such an association [13]. Gr/gr deletions are in fact a group of different deletions caused by recombination between the amplicons g1/g2, r1/r3 and r2/r4 [12], [14], [15].

The b2/b3 deletion removes 1.8 Mb of AZFc and appears to be a polymorphism without an obvious effect on fertility, despite the absence of a number of AZFc genes. The b2/b3 deletion is derived from a gr/rg inversion or b2/b3 inversion [10], [16]. The association of the b2/b3 partial deletion with male infertility has been reported in Chinese men, whereas no such a predisposition was detected in other populations [10], [16], [17], [18], [19], [20]. The b1/b3 deletion is rare and its frequency varies amongst populations with only 18 deletions published to date [12], [21]–[23]. Because this deletion has a much lower frequency than the gr/gr deletion, its effect on spermatogenesis is unkown [16].

Our objective was to determine the prevalence of different forms of *AZF* microdeletions, to evaluate their association with spermatogenic failure, and to define genetic association between the partial *AZFc* deletions in a well-defined case and control population.

## Materials and Methods

### Subjects

This study populations consisted of 339 men with spermatogenic failure and 256 normozoospermic men. All patients and controls were of Moroccan ancestry. Selection criteria were stringent; Ejaculates were obtained by masturbation after 2–7 days of sexual abstinence. All underwent an andrological work-up, which included medical history, physical examination, hormonal evaluation (FSH, LH, and testosterone) and semen analysis. Men with known clinical (cryptorchidism, infections, varicocele) or genetic (karyotype anomalies) causes of infertility were excluded. The normospermic men were selected from couples attending infertility clinics in the Casablanca region over a period of 12 years. These men had ≥20×10/ml sperm concentration, ≥40×106 total sperm count, ≥2 ml semen volume, ≥50% of a+b or ≥25% a motility, high percentage of normal forms (≥10%) with normal spermatogenesis according to WHO criteria. The infertility in these couples was due to female factors and/or these men had previously induced a spontaneous pregnancy in the current or a former relationship.

On the basis of repeated semen analyses and World Health Organization criteria the men with idiopathic spermatogenic failure were divided into 2 groups (Table 1). We obtained written informed consent from each subject and the study protocol was approved by the Committee on Research Ethics of Institut Pasteur du Maroc.

**Table 1:** Classification of case and control cohorts according to WHO criteria and incidence of Y chromosome microdeletions.

Group	Phenotype	N°	WHO criteria	Intact Y chromosome	AZF deletion (n)	gr/gr partial deletion	b2/b3 partial deletion	b1/b3 partial deletion	Partial deletion and associated Y chromosome haplogroup
**Severe**	non-obstructive azoospermia	88	no sperm in the ejaculate	80	AZFa (1), AZFc (3), AZFa+b (2), AZFa+b+c (2)	2 (2.5%)	-	1 (1.25%)	gr/gr, E3b2 n = 1; E3 (xE3b2), n = 1; b1/b3, E3b2 n = 1
	Oligozoospermia	12	<5×106 sperm/ml	11	AZFc (1)	-	-	-	
	Oligoasthenozoospermia	11	<5×106 sperm/ml and >40% of sperm have low motility	11	-	1 (9.09%)	-	-	gr/gr, F, n = 1
	Oligoteratozoospermia	7	<5×106 sperm/ml and >40% of sperm with abnormal morphology	7	-	-	-	-	-
	Oligoasthenoteratozoospermia	61	5×106 sperm/ml, >40% of sperm have low motility and >40% of sperm with abnormal morphology	57	AZFc (4)	6 (10.53%)	4 (7.02%)	-	gr/gr E3b2, n = 5 b2/b3 E3b2, n = 2 J, n = 1
**Mild**	Oligozoospermia	20	<20×106 sperm/ml	20	-	-	-	-	-
	Oligoasthenozoospermia	12	<20×106 sperm/ml and >40% of sperm have low motility	12	-	1 (8.33%)	-	-	-
	Oligoteratozoospermia	3	<20×106 sperm/ml and >40% of sperm with abnormal morphology	3	-	-	-	-	-
	Oligoasthenoteratozoospermia	22	<20×106 sperm/ml, >40% of sperm have low motility and >40% of sperm with abnormal morphology	22	-	2 (9.09%)	-	1 (4.54%)	gr/gr E3b2, n = 1 F, n = 1 b1/b3 E3b2, n = 1
	Asthenozoospermia	61	>40% of sperm have low motility	61	-	5 (8.19%)	-	-	gr/gr, E3b2 n = 4
	Teratozoospermia	19	>40% of sperm with abnormal morphology	19	-	1 (5.26%)	-	-	-
	Asthenoteratozoospermia	23	>40% of sperm have low motility and >40% of sperm with abnormal morphology	23	-	1 (4.34%)	-	-	-
Normospermic		256		256	-	16 (6.25%)	-	-	gr/gr E3b2, n = 9 E (xE3b2), n = 2 J, n = 1
**Total**		595		582	13	35	4	2	

Y chromosome haplogroup is indicated for 31 samples with partial AZFc deletions.

#### Y chromosome microdeletions analysis

Genomic DNA was extracted from peripheral leukocytes by standard means of proteinase K digestion followed by phenol–chloroform extraction and ethanol precipitation.

In order to confirm the specific relationship with spermatogenesis, we analysed the classical AZFa, AZFb and AZFc deletion in all patients and controls, two multiplex PCR systems (A and B) were carried out according to the European Academy of Andrology/European Molecular Genetics Quality Network (EAA/EMQN) guidelines [24]. Each of these subjects was tested for six AZF loci: the STS primers used for AZFa (sY84, sY86), AZFb (sY127, sY134) and AZFc (sY254, sY255). The internal control used was SRY14, samples from normal fertile men, without Y chromosome microdeletions and from healthy women, were used as normal controls, blank served as negative control.

The samples, in which the deletions were detected, were confirmed by repeating the PCR analysis at least three times.

#### Screening for partial AZFc deletions

Patients without classical AZFa, AZFb and AZFc microdeletions and all control subjects were screened for partial AZFc deletions. The presence or absence of the AZFc partial deletions in all subjects was tested by PCR using genomic DNA, amplifying STS markers [25]. The STSs were sY1191, sY1291, sY1206, sY1197, sY1258 and sY1201. A gr/gr deletion was identified by the absence of marker sY1291 and presence of other STSs. The b2/b3 deletions were characterized by the absence of the STS sY1191 and the presence of other STSs. The absence of markers sY1291, sY1191 and sY1197 combined with the presence of others indicated the b1/b3 deletion.

#### Y chromosome haplotypes analysis

Samples were genotyped for Y chromosome binary markers that are informative for North African populations. These were M35 (E3B), M81 (E3b2), 12f2a (J), M173 (R), and M89 (F). The Y genealogy used is based on that published by the Y Chromosome Consortium [26].

#### Statistical analysis

Differences among frequencies were calculated using the chi square (χ^2^)-test or Fisher's exact test, all p-values were based upon two-tailed tests and Odds ratios (ORs), 95% confidence intervals (CIs) were carried out, and P values<0.05 were taken as statistically significant.

## Results

### Screening for classical AZF microdeletions

In total, 13 out of the 339 infertile men (3.83%) with spermatogenesis impairment were found to have classical AZF deletions using STS analysis of genomic DNA (Table 1). These cases included one AZFa deletion, two AZFb+c deletions and two AZFa+b+c found in azoospermia patients and 8 AZFc deletions associated with 3 cases of azoospermia and 5 cases of severe oligozoospermia. No microdeletions were found in the normozoospermic controls.

### Screening for partial deletions of AZFc region

After excluding 13 patients with AZF deletions, we evaluated the distributions of partial AZFc deletions in 326 infertile patients and 256 normozoospermic men. We found three types of partial AZFc deletions in patients (Table 1); 19 (5.82%) gr/gr, 4 (1.22%) b2/b3 and 2 (0.61%) b1/b3 deletions. In contrast, screening of 256 control men revealed 16 (6.25%) gr/gr partial deletions and no b2/b3 nor b1/b3 deletions.

### Y chromosome haplogroups

Of the 41 patients carrying partial AZFc deletions sufficicent DNA was available for determining the Y chromosome haplogroup in 31 cases (Table 1). The distribution of haplogroups was not significantly different between the case and control populations. The high frequency of E3b2 chromosomes is consistent with previous studies of Moroccan populations where 80% of chromosome carry this marker [27], [28].

## Discussion

Classical AZF deletions, defined by a few well-designed markers [24], are clinically relevant because a clear cause–effect relationship between these deletions and spermatogenic failure has been established [29]–[32]. In this study, the overall frequency of AZF microdeletions was 3.83% in infertile men of Moroccan ancestry. This prevalence is similar to the first study in Moroccan population (3.15%) [33], and is consistent with the frequencies reported by other studies which range from 3% to 55% [1], [34], [35]. The prevalence of AZF microdeletions in patients with azoospermia and severe oligozoospermia was 9.09% and 5.49%, respectively. This incidence is similar to other reports describing a prevalence of AZF microdeletions in the range of 4.25% to 23% and 0.1% to 8.5% in patients with non-obstructive azoospermia and severe oligozoospermia respectively [36]–[38].

In our study only a single AZFa deletion was present and deletions were not detected in the AZFb region. Using an STS based approach we identified three types of partial AZFc deletion, gr/gr, b2/b3 and b1/b3. The gr/gr group of deletions was present in patient and control cohorts at similar frequencies of 5.83% and 6.25% respectively.. This result is similar to several studies that have not detected an association between the gr/gr deletion and failure of spermatogenesis [18], [19], [32], [39], [40]. The frequency of the gr/gr deletion varies on different Y chromosome backgrounds. The gr/gr deletion was initially observed in 14/43 different Y haplogroups suggesting that this deletion has occured several times during human evolution [12]. In one Y chromosome haplogroup that is common in men of Japanese origin, D2b, all samples studied carried the gr/gr deletion suggesting that the deletion is fixed in this haplogroup [12]. In our study the gr/gr deletions were present on different Y chromosome haplogroups indicating multiple deletion events and, in 80% of cases, it was present on the E3b2 haplogroup. Although this is suggestive of a high frequency of gr/gr deletion within this haplogroup, it should be noted that haplogroup E3b2 is present at high frequencies (80–85%) in the general North African population [27], [28]. It is important to note that gr/gr deletions are not a homogenous group of rearrangements within the AZFc region [25]. The various subclasses of gr/gr deletions show variation in gene loss and may be associated with other complex rearrangements such as duplications and inversions [25]. It is formally possible that the gr/gr deletions that we have identified in our case and control population may belong to distinct subclasses that could have an impact on spermatogenesis.

In contrast, the partial deletions b2/b3 and b1/b3 were present only in the infertile patient cohort. The b2/b3 deletion was found in 4 patients with severe oligoastheno-teratozoospermia, furthermore the b1/b3 deletion was presented in 2 patients, one with azoospermia and the other with oligoastheno-teratozoospermia. We have previously reported these deletions in the Moroccan population [33]. Including the data from our previous study we have now identified 6/475 infertile men carrying the b2/b3 deletion (p = 0.0318) and 2/475 infertile men carrying the b1/b3 deletion (p = 0.5006). These deletions were not observed in the 432 control samples.

The b2/b3 deletion was originally reported at high frequencies in northern Eurasian populations where it is fixed on the Y chromosome haplogroup N suggesting a founder mutation [16]. This haplogroup is the majority haplogroup in some Baltic populations and it does not appear to have an effect on reproductive fitness [16]. However, the b2/b3 deletion shows a strong association with idiopathic male infertility in the Han Chinese population [20]. These b2/b3 deletions were present on different Y chromosome haplogroups than the haplogroup N found in Northern Eurasian populations. This suggests that depending on the Y chromosome background the b2/b3 may have an adverse effect on spermatogenesis or that the b2/b3 deletions actually represent a family of closely related but distinct deletions that differ in their influence on spermatogenesis. Indeed, here we observed the b2/b3 deletion on Y chromosome haplogroups E3b2 and J.

Based on these observations we would recommend screening for b2/b3 deletions in larger cohorts of case and control populations of different ethnic/geographic origins to further understand the contribution of b2/b3 deletions to male infertility.
